# Organ Failure, Endotoxin Activity, and Mortality in Septic Shock

**DOI:** 10.1097/CCE.0000000000001308

**Published:** 2025-08-28

**Authors:** Luca Molinari, Mark A. Tidswell, Ali Al-Khafaji, Danielle Davison, Claude Galphin, Esha Kamaluddin, Debra M. Foster, John A. Kellum

**Affiliations:** 1 Department of Translational Medicine, Università degli Studi del Piemonte Orientale, Novara, Italy.; 2 Division of Pulmonary and Critical Care Medicine, Baystate Medical Center, Springfield, MA.; 3 Department of Critical Care Medicine, University of Pittsburgh, Pittsburgh, PA.; 4 Division of Critical Care Medicine, The George Washington University, Washington DC.; 5 Southeast Renal Research Institute, CHI Memorial Hospital, Chattanooga, TN.; 6 Spectral Medical, Toronto, ON, Canada.

**Keywords:** endotoxin, multiple organ failure, polymyxin B, sepsis, septic shock

## Abstract

**IMPORTANCE:**

The relationship between endotoxin activity, organ failure, and mortality is not well understood.

**OBJECTIVE:**

To test whether the combination of endotoxin activity and organ failure identifies patients at higher risk of death from sepsis and determine the relationship to previously described sepsis phenotypes.

**DESIGN, SETTING, AND PARTICIPANTS:**

Prospective observational study in four ICUs enrolling critically ill patients with septic shock.

**MAIN OUTCOMES AND MEASURES:**

Endotoxin activity assay (EAA) results, Sequential Organ Failure Assessment (SOFA), and multiple organ dysfunction (MODS) and 28-day mortality.

**RESULTS:**

We enrolled 90 patients aged 25–95 years and set an EAA cutoff of greater than or equal to 0.6 together with SOFA greater than 11 or MODS greater than 9 to define endotoxic septic shock (ESS). At baseline mean EAA was 0.64 (sd = 0.19), whereas mean SOFA and MODS were 10.3 (sd 3.2) and 5.8 (sd 3.1), respectively. EAA greater than or equal to 0.6 and SOFA greater than 11 were present in 20 patients (23.3%) and these patients had 60% mortality. EAA greater than or equal to 0.6 and SOFA less than or equal to 11 occurred in 31 (36.0%) with mortality 12.9%. Of the 35 remaining patients with EAA less than 0.6, 29 (33.7%) had SOFA less than or equal to 11 and 5 of them (17.2%) died. Only six patients (7.0%) had EAA less than 0.6 and SOFA greater than 11 and none died (*p* < 0.001). All patients with MODS greater than 9 also had EAA greater than or equal to 0.6 (12 patients) with 75% mortality. EAA greater than or equal to 0.6 with MODS less than or equal to 9 occurred in 39 patients with 17.9% mortality (*p* < 0.001). ESS (EAA ≥ 0.6 together with SOFA > 11 or MODS > 9) occurred in 21 patients and they had significantly higher mortality (57.1% vs. 15.9%, *p* < 0.001) compared with non-ESS, with a relative risk for death of 3.58 (95% CI, 1.86–6.91). Among ESS patients, 7 (33.3%) had δ phenotype, whereas only 4 (5.8%) had δ among non-ESS (*p* = 0.001).

**CONCLUSIONS AND RELEVANCE:**

ESS compromises patients with the highest mortality rate from sepsis. Such patients are most appropriate for trials testing anti-endotoxin therapy for improving survival.

**KEY POINTS**

**Question**: Can endotoxin activity assay (EAA) in combination with organ failure scores identify patients with higher severity septic shock as determined by observed mortality and clinical phenotype?

**Findings**: Among 90 critically ill patients with severe septic shock, we found that patients with endotoxic septic shock (ESS) (EAA ≥ 0.6 together with Sequential Organ Failure Assessment > 11 or multiple organ dysfunction > 9) had significantly higher 28-day mortality rate (57.1% vs. 13.8%) and higher rates of δ phenotype 33% vs. 5.8% compared with patients with other forms of septic shock.

**Meaning**: Patients with ESS present the highest risk of death and these patients may benefit from trials testing anti-endotoxin therapy.

High endotoxin activity may represent an endotype of septic shock (1, 2) and is the target for an ongoing clinical trial using hemoadsorption (3). However, risk of death from sepsis is known to be correlated with the number of organ systems failing and the severity of organ dysfunction (4). Low doses of endotoxin induce inflammatory cytokine production and sepsis-like physiology in normal humans (5, 6). In animals given lethal challenges with endotoxin, kidney and liver injury along with endothelial dysfunction are the most prominent manifestations in addition to circulatory shock (7). Similarly, in an unusual case report of self-administration of high-dose endotoxin, the patient developed profound shock together with acute kidney injury (AKI), liver dysfunction, and severe coagulopathy (8). This pattern of organ failure is characteristic of the δ phenotype described by Seymour et al (9) using machine learning and unsupervised clustering methods on clinical and laboratory data. The four phenotypes (α, β, γ, or δ) differ in patterns of organ dysfunction, prognosis, and response to treatment, δ has the highest mortality.

We conducted a prospective, multicenter, observational study to assess organ failure patterns in critically ill patients with septic shock and endotoxin activity using the endotoxin activity assay (EAA), a novel assay that can detect endotoxin in whole blood by use of neutrophil-dependent chemiluminescence. Our primary objective was to classify organ failures using Sequential Organ Failure Assessment (SOFA) (10) and Multiple Organ Dysfunction Score (MODS) (11) in patients with septic shock in relation to EAA levels. We prospectively defined endotoxic septic shock (ESS) (1, 2) as the combination of endotoxin activity greater than or equal to 0.6 units using EAA (corresponding to approximately 1000 pg/mL) (12) and evidence of significant organ failure using either SOFA greater than 11 or a MODS greater than 9 (13, 14) and examined 28-day mortality. Our secondary objective was to determine individual organ failures/dysfunctions and their relationship with EAA and ESS. Our third objective was to classify patients using a model of four clinical phenotypes for sepsis (9) and to examine the proportion of patients with ESS and the δ phenotype.

## MATERIAL AND METHODS

### Study Design and Patients

We performed a prospective, multicenter, observational study enrolling patients with septic shock in four ICUs in the United States from March 14, 2022 to December 7, 2023. Inclusion criteria were: 1) 18 years old or older, 2) known or suspected infection (fulfilling Sepsis-3 criteria) (15), and 3) hypotension requiring greater than 0.05 µg/kg/min norepinephrine or equivalent vasopressor support. We did not exclude patients enrolled in interventional trials including trials targeting endotoxin. Details about inclusion and exclusion criteria are reported in **Supplemental Appendix 1** (https://links.lww.com/CCX/B543). The study was conducted in accordance with the ethical standards of the responsible committee on human experimentation and with the Helsinki Declaration of 1975 and was approved by WCG institutional review board (IRB00000533) on August 25, 2021, and by individual institutions when required. Informed consent was collected from all patients or their surrogates. Our study complies with the Strengthening the Reporting of Observational Studies in Epidemiology Statement guidelines for reporting observational studies (**Supplemental Appendix 2**, https://links.lww.com/CCX/B543). We collected data including demographics, comorbidities, vital signs, organ dysfunction, and vasopressor use from enrollment/baseline (day 0) until day 3. All subjects were followed daily until day 3 and then for the first of either date of discharge alive from hospital or date of death truncated at 60 days. Based on a prior studies, we expected approximately one-third of patients with septic shock to have high endotoxin activity (13) and a mortality rate of around 60% and 25% for ESS and non-ESS, respectively (16). To identify this difference, we calculated a sample size of 100 patients in order to enroll at least 30 patients with high EAA.

### Endotoxin Activity Assay

We measured endotoxin activity in fresh whole blood using EAA (Spectral Medical, Toronto, ON, Canada) as per manufacturer’s instructions. EAA is the only Food and Drug Administration approved assay for endotoxin activity in blood. EAA measures the ability of lipopolysaccharide-antibody complexes to enhance the production of reactive oxygen species by neutrophils in a patients’ blood sample. The result is reported semiquantitatively as low EAA (< 0.4), intermediate (0.4–0.59), and high (≥ 0.6). A non-responder (NR) result occurs when the neutrophils in the sample are unable to respond adequately to the LPS challenge. Detailed descriptions about measurement, technique, and clinical significance of EAA are available in Supplemental Appendix 1 (https://links.lww.com/CCX/B543) and elsewhere (2, 12).

### Exposures and Outcomes

For our primary analysis we classified baseline SOFA as less than or equal to or greater than 11, MODS less than or equal to or greater than 9, and EAA less than or greater than or equal to 0.6. ESS was defined as a baseline EAA greater than or equal to 0.6 plus either SOFA greater than 11 or MODS greater than 9. We also calculated the mean values for SOFA, MODS, and EAA over days 1–3 (although ESS was classified only based on baseline values). Our primary outcome was 28-day mortality. Secondary outcomes were 60-day mortality and survival, both reported in the **Supplemental Appendix 3** (https://links.lww.com/CCX/B543). The choice of cutoff value 9 for MODS was based on previous evidence and the Evaluating the Use of Polymyxin BHemoperfusion in a Randomized Controlled trial of AdultsTreated for Endotoxemia and Septic Shock (EUPHRATES) trial (13, 14). MODS requires central venous pressure measurement, which in contemporary practice is often unavailable. A cutoff value 11 for SOFA score predicts a similar in-hospital mortality to MODS score of 9 (approximately 50%) (10, 11, 17).

We determined the severity of shock (vasopressor dose expressed as norepinephrine equivalents [18]) as well as the other types of organ dysfunction such as AKI (according to serum creatinine criteria from Kidney Disease Improving Global Outcomes (KDIGO) (19) and excluding patients with end-stage renal disease), respiratory dysfunction and severe respiratory dysfunction/failure (use of mechanical ventilation), neurologic dysfunction (Glasgow Coma scale ≤ 9 [20]), hepatic dysfunction (bilirubin ≥ 2 mg/dL), and hematologic dysfunction (platelet count < 100,000/mcL). Additional details about organ dysfunctions definitions are provided in **Supplemental Appendix 4** (https://links.lww.com/CCX/B543).

We collected the necessary clinical data and assigned each patient a clinical phenotype based on the model developed by Seymour et al (9) which uses baseline clinical information to determine one of four phenotypes (α, β, γ, and δ). This model used unsupervised clustering methods based clinical variables associated with sepsis. We assigned a phenotype to each patient by calculating the Euclidean distance from each patient to the centroid of each phenotype from the derivation cohort as previously described (21).

### Statistical Analysis

We reported mean (sd) values for SOFA, MODS, and EAA and patients were classified in four groups according to the different combinations of EAA (< 0.6/≥ 0.6) and SOFA (≤ 11/> 11) or MODS (≤ 9/> 9). Mortality in patients with ESS was compared with the rest non-ESS patients using Pearson’s chi-square test and relative risk with its 95% CI. We also performed logistic regression models with 28-day mortality as dependent variable to report unadjusted and adjusted odds ratios (ORs) for ESS. The adjusted model used age, sex, race, and Elixhauser index as covariates. To assess 60-day survival, we used Cox proportional hazard model to report hazard ratio (HR) for ESS after adjusting for the same covariates used above. We reported adjusted survival curves. The statistical analysis for baseline EAA greater than or equal to 0.6, SOFA greater than 11 and MODS greater than 9 and their Cox model and 60-day survival curves are reported in Supplemental Appendix 3 (https://links.lww.com/CCX/B543).

A sensitivity analysis for the primary endpoint was restricted to patients also meeting the lactate criteria (> 2mmol/L) for septic shock (**Supplemental Appendix 5**, https://links.lww.com/CCX/B543). A second sensitivity analysis explored different SOFA and MODS cutoffs comparing each by sensitivity and specificity for mortality. We used the Youden index for comparisons across cutoffs (**Supplemental Appendix 6**, https://links.lww.com/CCX/B543).

In Supplemental Appendix 4 (https://links.lww.com/CCX/B543), we reported the details of the statistical analysis regarding SOFA/MODS/EAA mean over days 1–3 compared with their baseline values and as well the analysis regarding severity of shock, AKI, and the other different organ dysfunctions.

We reported baseline EAA/SOFA/MODS and the distribution of EAA greater than or equal to 0.6/SOFA greater than 11/MODS greater than 9, ESS and 28-day mortality across the four phenotypes. We compared proportions of patients exhibiting δ phenotype between patients with ESS and other forms of septic shock using the chi-square test. We used SPSS Statistics Version 26 (IBM Corp., Armonk, NY) and the per-comparison significance was set at a two-tailed *p* value of less than 0.05. For the assignment of the phenotypes, we used R studio (version 4.4.0; R Core Team 2024, Vienna, Austria) with missRanger library.

## RESULTS

We enrolled 90 patients, age ranging from 25 to 95 years (mean age 65), of which 48 (53.3%) were male and 66 (73.3%) were Whites. Baseline clinical characteristics are shown in **Table 1**. On the day of enrollment (day 0, baseline), the mean SOFA score was 10.3 (sd 3.2), the mean MODS was 5.8 (sd 3.1) and mean EAA was 0.64 (sd 0.2). Overall, 23 (25.6%) patients died by day 28.

**TABLE 1.** Clinical Characteristics

Characteristics	Baseline (n = 90)	Day 1 (n = 90)	Day 2 (n = 85)^f^	Day 3 (n = 79)^g^
Age (yr)	65 (14)			
Sex				
Male	48 (53.3%)			
Female	42 (46.7%)			
Race				
White	66 (73.3%)			
Black	16 (17.8%)			
Hispanic	3 (3.3%)			
Other^a^	5 (5.6%)			
Weight (kg)	86.1 (23.9)			
Elixhauser Index	11 (9)			
Lactate (mmol/L)^b^	1.9 (1.3–3.2)	1.5 (1.0–2.6)	1.3 (1.0–2.0)	1.3 (1.0–2.0)
Lactate > 2.0 mmol/L^c^	53 (58.9%)			
C-reactive protein (mg/L)^d^	190 (96–280)	181.5 (81–279]	101 (53–196)	67 (39–147)
Vasopressor use (%)	90/90 (100%)	78/90 (86.7%)	49/85 (57.6%)	35/79 (44.3%)
Vasopressor dose^e^	0.2 (0.1–0.33)	0.1 (0.04–0.24)	0.02 (0.0–0.1)	0.0 (0.0–0.08)
End-stage renal disease	8/90 (8.9%)	8/90 (8.9%)	7/85 (8.2%)	7/79 (8.9%)
Any acute kidney injury (%)	41/90 (45.6 %)	37/90 (41.1%)	34/85 (40.0%)	27/79 (34.2%)
KDIGO stage 1	9/90 (10.0%)	9/90 (10.0%)	10/85 (11.8%)	6/79 (7.6%)
KDIGO stage 2	11/90 (12.2%)	6/90 (6.7%)	5/85 (5.9%)	7/79 (8.9%)
KDIGO stage 3	21/90 (23.3%)	22/90 (24.4%)	19/85 (22.4%)	14/79 (17.7%)
Mechanical ventilation (%)	18/90 (20%)	17/90 (18.9%)	16/85 (18.8%)	12/79 (15.2%)
Sequential Organ Failure Assessment score	10.3 (3.2)	9.3 (4.1)	7.9 (4.4)	7 (4.8)
Multiple Organ Dysfunction Score	5.8 (3.1)	5.3 (3.6)	4.8 (3.3)	4.5 (3.7)
EAA	0.64 (0.19)	0.64 (0.21)	0.63 (0.31)	0.58 (0.25)
EAA ≥ 0.6 (%)	51/90 (56.7%)	56/90 (62.2%)	49/81 (60.5%)	36/75 (48%)
EAA non-responder (%)	4/90 (4.4%)	2/90 (2.2%)	0/81 (0%)	0/75 (0%)
Mortality at 28 d	23 (25.6%)			
Mortality at 60 d	25 (27.8%)			

EAA = endotoxin activity assay, KDIGO = Kidney Disease Improving Global Outcomes.

a Unknown/declined.

b Missing data for 2 patients at baseline, for 5 at day 1, for 8 at day 2, and for 15 at day 3.

c Patients with maximum lactate over days 0 (baseline) to 3 > 2.0 mmol/L.

d Missing data for 8 patients at baseline, for 8 at day 1, for 9 at day 2, and for 15 at day 3.

e Maximum daily dose in norepinephrine equivalents in µg/kg/min.

f Missing data from five patients, four died, and one was discharged alive.

g Missing data from 11 patients, 7 died, and 4 were discharged alive.

Continuous variables are expressed as means (sd) or medians (interquartile range) as appropriate. Categorical variables are expressed as absolute numbers (percentage).

### Baseline Organ Failure and EAA in Relation to Mortality/Survival

**Figure 1.**
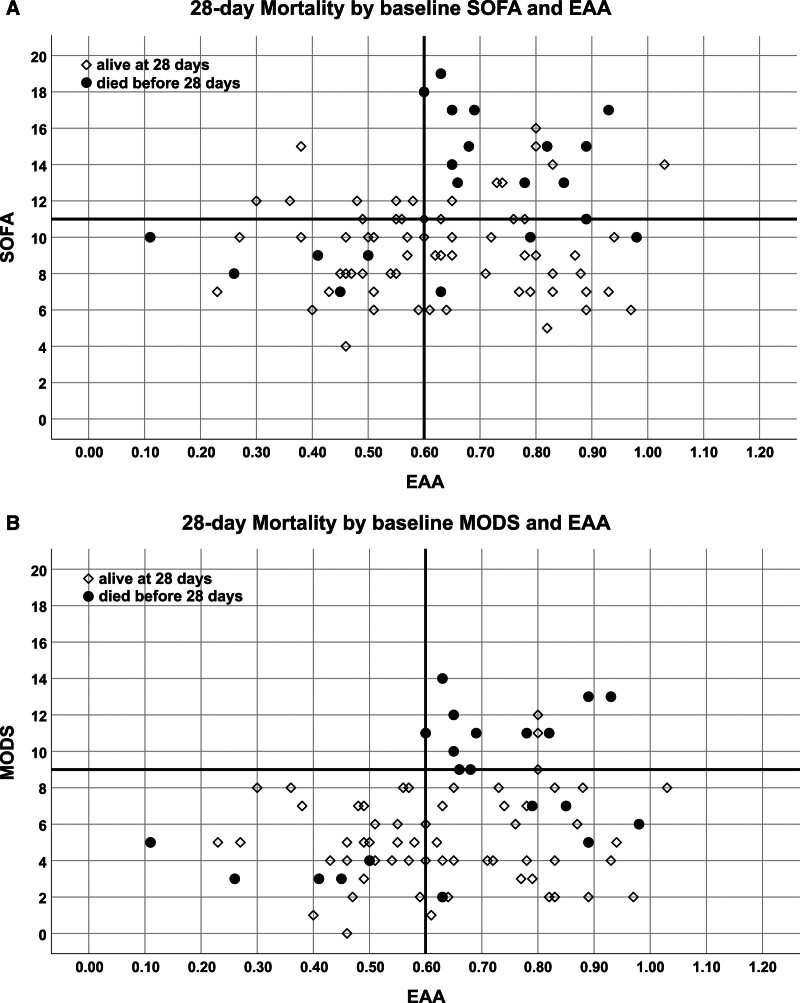
Each patient is represented by a *dot/square* according to his baseline (day 0) values for endotoxin activity assay (EAA) and organ failure scores (Sequential Organ Failure Assessment [SOFA] [**A**]; and Multiple Organ Dysfunction Score [MODS] [**B**]). *Full black circles* represent patients who died, whereas *empty squares* represent patients alive at 28 days (or discharged alive before day 28). *Solid black lines* represent the cutoff values of 11 for SOFA (**A**), of 9 for MODS (**B**) and of 0.60 for EAA.

The relationship between baseline EAA and SOFA and the corresponding 28-day mortality are shown in **Figure 1*A***; the relationship with MODS is shown in **Figure 1*B***. **Supplemental Figure 1** (https://links.lww.com/CCX/B543) depicts these relationships for 60-day mortality. SOFA greater than 11 was present in 27 (30%) patients and 13 (48%) of these patients died by day 28. By contrast, there were only 10 (15.9%) deaths among the 63 patients with SOFA less than or equal to 11. Higher mortality (75%) was observed for the 12 patients (13.3% of all) with MODS greater than 9. Whereas of the remaining 78 patients with MODS less than or equal to 9, only 14 (17.9%) died. Mortality by day 28 occurred in 5 of 35 (14.3%) patients with a baseline EAA less than 0.6 while 16 of 51 (31.4%) died with EAA greater than or equal to 0.6. Four patients (4.4%) were NR for EAA and mortality was 50% for these patients.

EAA greater than or equal to 0.6 and SOFA greater than 11 were present together in 20 patients (23.3% of valid test results) and 12 (60%) of these patients died by day 28. By contrast EAA greater than or equal to 0.6 and SOFA less than or equal to 11 occurred in 31 (36.0%), and 4 (12.9%) died. Of the 35 remaining patients with EAA less than 0.6, 29 (33.7%) had SOFA less than or equal to 11 and 5 (17.2%) died. Only six patients (7.0%) had low EAA and high SOFA and none died (overall *p* < 0.001). Results were qualitatively similar using MODS although all patients with MODS greater than 9 also had EAA greater than or equal to 0.6 (12 patients) and 28-day mortality was 75%. High EAA with MODS less than or equal to 9 occurred in 39 (45.3%) patients, 7 (17.9%) of them died by day 28. Only 5 (14.3%) patients died among the 35 with low MODS and EAA (overall *p* < 0.001). ESS (EAA greater than or equal to 0.6 and high organ failure using either SOFA greater than 11 or MODS > 9) was present in 21 patients (23.3%) and compared with remaining patients with other forms of septic shock (non-ESS), those with ESS had significantly higher mortality (57.1% vs. 15.9%, *p* < 0.001) with relative risk for death of 3.58 (95% CI, 1.86–6.91); **Supplemental Table 1** (https://links.lww.com/CCX/B543) summarizes these results. The logistic regression model for 28-day mortality reported for ESS unadjusted OR of 7.03 (95% CI, 2.39–20.66; *p* < 0.001) and, after adjusting for age, sex, race, and comorbidity (Elixhauser index), an adjusted OR of 8.56 (95% CI, 2.58–28.41; *p* < 0.001).

**Figure 2.**
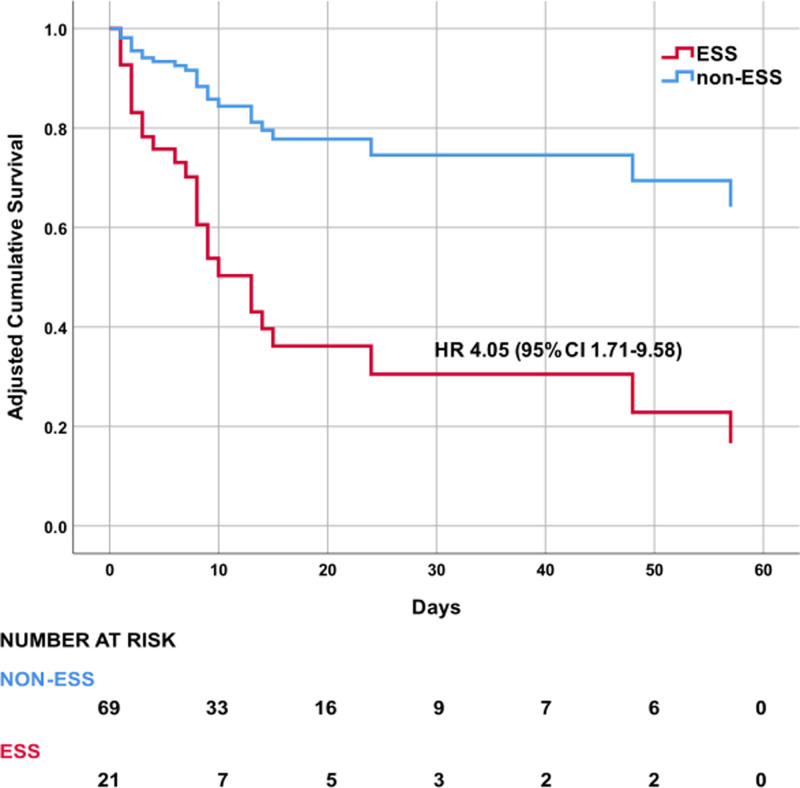
Shown are the adjusted 60-day survival curves obtained from Cox proportional hazard model for patients with and without endotoxic septic shock (ESS). ESS was defined as the presence of baseline endotoxin septic shock greater than or equal to 0.6 together with either baseline Sequential Organ Failure Assessment greater than 11 or multiple organ dysfunction greater than 9. Patients not meeting these criteria were classified as non-ESS. Numbers of patients at risk of death are shown beneath the figure. The model was adjusted for age, sex, race, and Elixhauser index (for burden of comorbidities). HR = hazard ratio.

**Figure 2** depicts the adjusted 60-day survival curves for ESS and non-ESS patients. The HR for ESS was 4.05 (95% 1.71–9.58, *p* = 0.001). The HRs and adjusted 60-day survival curves for baseline EAA greater than or equal to 0.6, SOFA greater than 11 and MODS greater than 9 are reported in Supplemental Appendix 3 and **Supplemental Figures 4–6** (https://links.lww.com/CCX/B543).

In the first sensitivity analysis we repeated the primary analysis for patients who also met full Sepsis-3 criteria (including serum lactate > 2mmol/L) for septic shock. In this subset of 53 patients ESS mortality as 64.7% vs. 19.4% for non-ESS, (*p* = 0.001) with relative risk for death of 3.33 (95% CI, 1.57–7.06) (see Supplemental Appendix 5 and Supplemental Table 1 for additional details, https://links.lww.com/CCX/B543).

Regarding the second sensitivity analysis for alternative cutoffs for SOFA and MODS, details are provided in Supplemental Appendix 6 (https://links.lww.com/CCX/B543). Briefly, the best cutoffs for predicting 28-day mortality were SOFA greater than 12 and MODS greater than 8. Using these two to define ESS led us to similar results with 20 patients (22.2%) meeting ESS criteria and ESS having again significantly higher mortality (60% vs. 15.7%, *p* < 0.001) with a comparable relative risk of 3.82 (95% CI, 1.99–7.31).

### Organ Failure Scores and EAA at Baseline and Over Days 1–3 and Mortality

**Supplemental Figure 2** (https://links.lww.com/CCX/B543) presents the alluvial plots showing the relationship between EAA (2A), SOFA (2B), and MODS (2C) over baseline and days 1–3 with 28-day mortality. **Supplemental Figure 3** (https://links.lww.com/CCX/B543) depicts the same relationship for 60-day mortality. The distributions between individual organ failures, δ phenotype and ESS are shown in **Table 2**. Additional detailed results for each organ system are reported in Supplemental Appendix 4 (https://links.lww.com/CCX/B543). In Supplemental Appendix 4 (https://links.lww.com/CCX/B543) we also reported the full results comparing the changes of EAA, SOFA, and MODS from baseline to days 1–3.

**TABLE 2.** Organ System Failures in Endotoxic vs. Non-Endotoxic Septic Shock

Organ System	ESS^a^ (%)	Non-ESS	p
Lactate > 2.0 mmol/L^b^	17/21 (81.0%)	36/69 (52.2%)	0.02
AKI (any stage)	16/19 (84.2%)	32/63 (50.8%)	0.01
AKI (stages 2–3)	13/19 (68.4%)	21/63 (35.6%)	0.02
Respiratory dysfunction^c^	21/21 (100%)	48/69 (69.2%)	0.004
Mechanical ventilation	17/21 (81%)	10/69 (14.5%)	< 0.001
Neurologic dysfunction	18/21 (85.7%)	25/69 (36.2%)	< 0.001
Hepatic dysfunction	12/21 (57.1%)	15/69 (21.7%)	0.002
Hematologic dysfunction	15/21 (71.4%)	18/69 (26.2%)	< 0.001
Delta phenotype	7/21 (33.3%)	4/69 (5.8%)	0.001

AKI = acute kidney injury, ESS = endotoxic septic shock.

a Endotoxic septic shock was defined as a baseline endotoxin activity assay ≥ 0.6 plus either Sequential Organ Failure Assessment > 11 or Multiple Organ Dysfunction Score > 9.

b Patients with maximum lactate over days 0 (baseline) to 3 > 2.0 mmol/L.

c PO_2_/Fio_2_ < 300.

### Phenotypes

Finally, the distribution among our patients for the α, β, γ, and δ phenotypes, as described by Seymour (9), and their associated baseline EAA, SOFA, and MODS and mortality status are shown in **Supplemental Table 2** (https://links.lww.com/CCX/B543). Our hypothesis was that the proportion of patients with the δ phenotype would be greater among patients with ESS compared with remaining patients. Although there were only 11 patients with the δ phenotype, 7 (63.6%) had ESS. Conversely, ESS was seen in only 14 of 79 (17.7%) patients with other phenotypes. Therefore, among the 21 patients with ESS, 7 (33.3%) had the δ phenotype while among the 69 patients with non-ESS, only 4 (5.8%) had δ phenotype (*p* = 0.001) (Table 2). Excluding NR EAA results, mean baseline EAA was 0.73 (sd 0.16) for the δ phenotype compared with 0.62 (sd 0.19) for all others, mean difference 0.11 (95% CI, –0.02 to 0.24; *p* = 0.091).

## DISCUSSION

An ongoing randomized trial testing endotoxin removal with polymyxin B hemoadsorption uses both EAA and organ failure scores as enrollment criteria (NCT03901807). We sought to test the hypothesis that the combination of endotoxin activity and SOFA or MODS using predefined cutoffs, would identify patients at highest risk of death from sepsis. Our results indicate that ESS, defined by the combination of EAA greater than or equal to 0.6 and high organ dysfunction scores (SOFA > 11 or MODS > 9) identifies patients with the highest risk of death from sepsis with a relative risk of 3.58 (95% CI, 1.86–6.91) compared with non-ESS patients. Our cutoff for EAA is based on the analytic specifications of the assay and corresponds to approximately 1000 pg/mL (22). In critically ill humans, this threshold defines a high-risk category for developing sepsis-associated organ failure, a condition previously referred to as severe sepsis (23). Our cutoffs for SOFA and MODS, whereas prospectively defined, were more arbitrary. The median MODS in the EUPHRATES trial (13) was nine before an amendment that restricted enrollment to patients with MODS greater than 9. Based on published literature (24) and on comparing the scores themselves, a SOFA score of 11 should be roughly equivalent to MODS of 9. However, in our patient cohort the mean SOFA score was 10.3 while the mean MODS was 5.8. Applying Youden index, our sensitivity analyses suggest that SOFA scores of 12 and MODS of 8 are optimal for predicting mortality in septic shock with EAA greater than or equal to 0.6, but these cutoffs yielded similar results to our predefined thresholds.

However, retrospective real-world data from Japan indicate that endotoxin removal using hemoadsorption is effective in patients with SOFA scores as low as 7, although efficacy was greatest for SOFA scores between 10 and 12 (25). It is possible that this retrospective ascertainment of organ failure severity is less sensitive compared with prospective data collection. Or, although the performance of SOFA score to predict 28-day mortality (the most frequently used outcome in sepsis trials) is crucial for study design, SOFA may not fully capture the broader range of clinical benefits in the real-world setting.

Our results are qualitatively similar to those reported by Payen et al (26) in an analysis using HPLC/mass spectroscopy for detection of 3‑hydroxy myristate (3HM) to estimate endotoxin burden. Their results indicated that high endotoxin together with SOFA greater than 8 was associated with significantly greater mortality than either parameter by itself. However, endotoxin concentrations were low compared with prior studies using 3HM and the assay is not available clinically (27).

The pattern of organ failure may also be important in the selection of patients for anti-endotoxin therapy. Using a nation-wide database in Japan, Fujimori found that patients with respiratory, cardiovascular, hepatic and renal SOFA scores of two or more benefited from polymyxin B hemoadsorption whereas abnormalities in neurologic functions and coagulation were not predictive (25). Conversely, in a secondary analyses using data from the EUPHRATES trial (13) together with a large sepsis registry in Japan, Osawa found that patients with high lactate and coagulopathy appeared to benefit most from polymyxin B hemoadsorption (28). Taking both observations together, we expected to have many patients meeting ESS criteria also meet criteria for the δ phenotype of sepsis, as described by Seymour et al (9). The δ phenotype is characterized by more AKI, hepatic dysfunction, and coagulopathy. In our cohort, the majority (63.6%) of the 11 patients with the δ phenotype had ESS. Although significantly greater than for other phenotypes, ESS was still seen in nearly 20% of patients with the other phenotypes. Therefore, EAA testing would still be required to exclude ESS even if clinical phenotyping was used. Importantly, a number of clinical trials using antibodies to endotoxin have failed to demonstrate clinical benefit. Although numerous reasons have been suggested (29), one major problem was that evidence of endotoxemia was not an inclusion requirement. Our results suggest that only about one in four patients with septic shock are likely to benefit from therapies directed against endotoxin. Reasons for this clinical heterogeneity are unclear but differences in host-response and possibly intestinal microbiome across individuals may contribute (30).

An important limitation to our study concerns limited sample-size for subgroup analysis. In addition, we did not require positive cultures but rather, a clinical diagnosis of infection. Prior studies have shown no correlation between culture results or source of infection and EAA levels. This is likely because the primary source of endotoxin is translocation from the gut rather than from the infecting organism. Finally, we used EAA to quantify endotoxin activity rather than direct measures of endotoxin concentration (e.g., limulus amebocyte lysate, 3HM). However, no direct measures of endotoxin concentration in blood are clinically available and as such, EAA is the only approved test.

## CONCLUSIONS

ESS defined by the combination of endotoxin activity greater than or equal to 0.6 and high organ dysfunction scores (SOFA > 11 or MODS > 9) identifies patients with the highest risk of death from sepsis. Such patients are most appropriate for trials testing anti-endotoxin therapy for improving survival. Most patients with δ phenotype of sepsis had ESS.

## ACKNOWLEDGMENTS

The authors thank the clinical coordinators at the trial sites for their hard work in collecting the data used in this study. We also thank the patients and their families for consenting to this study. We also thank Jason N. Kennedy for the guidance provided in the phenotyping process.

## Supplementary Material
