# Supplementary material for: Organ Failure, Endotoxin Activity, and Mortality in Septic Shock
Source: Crit Care Explor. 2025 Aug 28;7(9):e1308. doi: 10.1097/CCE.0000000000001308 (PMC12398369; doi:10.1097/CCE.0000000000001308)
Supplement: Supplementary file 1 [file cc9-7-e1308-s001.pdf]

SUPPLEMENTAL DIGITAL CONTENT

Title: Organ Failure, Endotoxin Activity, and Mortality in Septic Shock

Authors:

Luca Molinari MD,[1] Mark A Tidswell MD,[2] Ali Al-Khafaji MD, MPH,[3] Danielle Davison MD,[4] Claude Galphin MD,[5] Esha Kamaluddin HBSc,[6] Debra M Foster BSc,[6] John A Kellum MD[3,6]

[1] Department of Translational Medicine, Università degli Studi del Piemonte Orientale, Novara, Italy

[2] Division of Pulmonary and Critical Care Medicine, Baystate Medical Center, Springfield MA, USA

[3] Department of Critical Care Medicine, University of Pittsburgh, Pittsburgh, PA, USA

[4] Division of Critical Care Medicine, The George Washington University, Washington D.C., USA

[5] Southeast Renal Research Institute, CHI Memorial Hospital, Chattanooga, TN, USA

[6] Spectral Medical Inc. Toronto, Ontario, Canada

INDEX OF CONTENTS:

SUPPLEMENTAL APPENDIX 1 – PATIENTS AND ENDOTOXIN ACTIVITY ASSAY 4
Patients 4
Inclusion Criteria 4
Exclusion Criteria 4
Endotoxin activity assay 5
SUPPLEMENTAL APPENDIX 2 - STROBE STATEMENT CHECKLIST 6
SUPPLEMENTAL APPENDIX 3 - MORTALITY AND SURVIVAL AT 60 DAYS 8
Statistical analysis 8
Results 8
SUPPLEMENTAL APPENDIX 4 - INDIVIDUAL ORGAN SYSTEM FAILURES AND EAA 9
Material and Methods 9
SOFA/MDOS/EAA at baseline and over days 1-2-3 in relation to 28-day mortality 9
Severity of shock 9
Acute Kidney Injury 9
Respiratory dysfunction 10
Neurological dysfunction 10
Hepatic dysfunction 10
Hematological dysfunction 10
Results 12
SOFA/MDOS/EAA at baseline and over days 1-2-3 in relation to 28-day mortality 12
Severity of shock 12
Acute Kidney Injury 12
Respiratory Dysfunction 13
Neurological dysfunction 14
Hepatic dysfunction 14
Hematological dysfunction 15
SUPPLEMENTAL APPENDIX 5 – SENSITIVITY ANALYSIS 1 – LACTATE > 2.0 16
Methods 16

Results 16
SUPPLEMENTAL APPENDIX 6 – SENSITIVITY ANALYSIS 2 – ALTERNATIVE CUTOFFS 17
Statistical analysis 17
Results 17
SUPPLEMENTAL TABLE 1 – SENSITIVITY ANALYSIS FOR LACTATE >2.0 19
SUPPLEMENTAL TABLE 2 – CLINICAL PHENOTYPES FOR SEPSIS 20
SUPPLEMENTAL FIGURE 1 - BASELINE EAA, SOFA/MODS AND 60-DAY MORTALITY 21
SUPPLEMENTAL FIGURE 2 – ALLUVIAL PLOTS FOR 28-DAY MORTALITY 22
SUPPLEMENTAL FIGURE 3 – ALLUVIAL PLOTS FOR 60-DAY MORTALITY 23
SUPPLEMENTAL FIGURE 4 – ADJUSTED 60-DAY SURVIVAL BY BASELINE EAA 24
SUPPLEMENTAL FIGURE 5 – ADJUSTED 60-DAY SURVIVAL BY BASELINE SOFA 25
SUPPLEMENTAL FIGURE 6 – ADJUSTED 60-DAY BY BASELINE MODS 26
SUPPLEMENTAL MATERIAL REFERENCES 27

SUPPLEMENTAL APPENDIX 1 – PATIENTS AND ENDOTOXIN ACTIVITY ASSAY

Patients

Inclusion Criteria

- 18 years of age or older
- Hypotension requiring vasopressor support: Requirement for at least one of the vasopressors listed below, at the dose shown below, for at least 2 continuous hours and no more than 30 hours:
  - Norepinephrine > 0.05mcg/kg/min
  - Dopamine > 10 mcg/kg/min
  - Phenylephrine > 0.4 mcg/kg/min
  - Epinephrine > 0.05 mcg/kg/min
  - Vasopressin > 0.03 units/min
  - Vasopressin (any dose) in combination with another vasopressor listed above (at any dose)
- Documented or suspected infection defined as definitive or empiric intravenous antibiotic administration and respecting Sepsis-3 criteria for sepsis (1). We did not require that patients met lactate criteria for septic shock because patients were enrolled from the ICU after therapy had already been initiated and pre-resuscitation values were not available. However, we collected data on lactate and conducted sensitivity analysis (see Supplemental Appendix 5) using the full Sepsis-3 criteria for septic shock.

Exclusion Criteria

- Lack of commitment for full medical support (i.e., patients with do not intubate or do not resuscitate orders, patients eligible or who have requested only comfort measures or palliative care).
- Inability to achieve or maintain a minimum mean arterial pressure (MAP) of ≥ 65 mm Hg despite vasopressor therapy and fluid resuscitation deemed adequate by the treating physician.
- There is clinical support for non-septic shock such as:
  - Acute pulmonary embolus
  - Transfusion reaction
  - Acute coronary syndrome with low cardiac output
- Severe granulocytopenia (leukocyte count less than 500 cells/mm3).

We did not exclude patients enrolled in interventional trials, including trials targeting endotoxin. In detail, 8 patients were co-enrolled in the TIGRIS trial (Polymyxin-B hemoadsorption NCT03901807), and 5 of them received at least some treatment.

Endotoxin activity assay

The Endotoxin activity assay (EAA) is a homogeneous assay for measuring the ability of LPS-antibody complexes to enhance the production of reactive oxygen species by neutrophils in a patients' blood sample. The key reagent is an IgM antibody that binds to lipid A of endotoxin. Whole blood collected in an EDTA anticoagulant collection tube is divided into 3 test tubes. Tube 1 measures the patient's baseline whole blood neutrophil activation and Tube 2 contains the IgM antibody which measures the patient's intrinsic endotoxin concentration and Tube 3 is a positive maximal exogenous endotoxin assay tube. The quantification of endotoxin activity is captured in a luminometer (Berthold Technologies GmbH & Co.KG, Bad Wildbad, Germany) and calculated as a ratio of the sample chemiluminescence minus the background divided by the signal in the maximum calibrator minus the background. Results are reported semi-quantitatively as low EAA (<0.4), intermediate (0.4-0.59) and high (≥0.6). There is a built-in fail safe such that a non-responder result (NR) occurs when there is <15% difference in reactive light units between tubes 2 and 3. This occurs when the neutrophils in the sample are unable to respond adequately to the LPS challenge in tube 3 and thus is interpreted as a non-reactive.

SUPPLEMENTAL APPENDIX 2 - STROBE STATEMENT CHECKLIST

	Item No	Recommendation	Page No
**Title and abstract**	1	(*a*) Indicate the study's design with a commonly used term in the title or the abstract	2
		(*b*) Provide in the abstract an informative and balanced summary of what was done and what was found	2
**Introduction**			
Background/rationale	2	Explain the scientific background and rationale for the investigation being reported	4
Objectives	3	State specific objectives, including any prespecified hypotheses	4
**Methods**			
Study design	4	Present key elements of study design early in the paper	4,5
Setting	5	Describe the setting, locations, and relevant dates, including periods of recruitment, exposure, follow-up, and data collection	5
Participants	6	(*a*) *Cohort study*—Give the eligibility criteria, and the sources and methods of selection of participants. Describe methods of follow-up<br>*Case-control study*—Give the eligibility criteria, and the sources and methods of case ascertainment and control selection. Give the rationale for the choice of cases and controls<br>*Cross-sectional study*—Give the eligibility criteria, and the sources and methods of selection of participants	5/Appendix 1
		(*b*) *Cohort study*—For matched studies, give matching criteria and number of exposed and unexposed<br>*Case-control study*—For matched studies, give matching criteria and the number of controls per case	NA
Variables	7	Clearly define all outcomes, exposures, predictors, potential confounders, and effect modifiers. Give diagnostic criteria, if applicable	5-6-7 /Appendix 3-4
Data sources/ measurement	8*	For each variable of interest, give sources of data and details of methods of assessment (measurement). Describe comparability of assessment methods if there is more than one group	6-7/Appendix 3-4
Bias	9	Describe any efforts to address potential sources of bias	7
Study size	10	Explain how the study size was arrived at	6
Quantitative variables	11	Explain how quantitative variables were handled in the analyses. If applicable, describe which groupings were chosen and why	7
Statistical methods	12	(*a*) Describe all statistical methods, including those used to control for confounding	7/Appendix 3-4
		(*b*) Describe any methods used to examine subgroups and interactions	7/Appendix 3-4
		(*c*) Explain how missing data were addressed	7
		(*d*) *Cohort study*—If applicable, explain how loss to follow-up was addressed<br>*Case-control study*—If applicable, explain how matching of cases and controls was addressed<br>*Cross-sectional study*—If applicable, describe analytical methods taking account of sampling strategy	NA
		(*e*) Describe any sensitivity analyses	7/Appendix 5-6

Continued on next page

**Results**			
Participants	13*	(a) Report numbers of individuals at each stage of study—eg numbers potentially eligible, examined for eligibility, confirmed eligible, included in the study, completing follow-up, and analysed	8
		(b) Give reasons for non-participation at each stage	NA
		(c) Consider use of a flow diagram	NA
Descriptive data	14*	(a) Give characteristics of study participants (eg demographic, clinical, social) and information on exposures and potential confounders	8/Table1
		(b) Indicate number of participants with missing data for each variable of interest	Table1
		(c) *Cohort study*—Summarise follow-up time (eg, average and total amount)	Appendix3
Outcome data	15*	*Cohort study*—Report numbers of outcome events or summary measures over time	8-9
		Case-control study—Report numbers in each exposure category, or summary measures of exposure	
		Cross-sectional study—Report numbers of outcome events or summary measures	
Main results	16	(*a*) Give unadjusted estimates and, if applicable, confounder-adjusted estimates and their precision (eg, 95% confidence interval). Make clear which confounders were adjusted for and why they were included	8-9/Appendix 3-4
		(*b*) Report category boundaries when continuous variables were categorized	
		(*c*) If relevant, consider translating estimates of relative risk into absolute risk for a meaningful time period	
Other analyses	17	Report other analyses done—eg analyses of subgroups and interactions, and sensitivity analyses	9-10/ Appendix 5-6
**Discussion**			
Key results	18	Summarise key results with reference to study objectives	11-12
Limitations	19	Discuss limitations of the study, taking into account sources of potential bias or imprecision. Discuss both direction and magnitude of any potential bias	12-13
Interpretation	20	Give a cautious overall interpretation of results considering objectives, limitations, multiplicity of analyses, results from similar studies, and other relevant evidence	13
Generalisability	21	Discuss the generalisability (external validity) of the study results	13
**Other information**			
Funding	22	Give the source of funding and the role of the funders for the present study and, if applicable, for the original study on which the present article is based	1

SUPPLEMENTAL APPENDIX 3 - MORTALITY AND SURVIVAL AT 60 DAYS

The primary outcome was 28-day mortality and it is reported in the main text. In this Supplemental Appendix 3 we reported the methodology and results related to 60-day mortality and survival up to 60 days from enrollment.

Statistical analysis

All patients were followed daily to day 3, then for the first of either date of discharge alive or date of death truncated at 60 days following the date of informed consent/enrollment.

We reported the relationship between baseline EAA, SOFA/MODS and mortality status at 60 days using a dot plot figure (Supplemental Figure 1) similarly to what we did in the main text with mortality at 28 days and Figure 1.

We assessed adjusted 60-day survival using Cox proportional hazard models. For the Cox model, 60-day survival was the dependent variable while the covariates of interest were baseline EAA ≥0.6, SOFA >11, MODS >9. The model for ESS is already reported in the main text and in Figure 2. All the models were adjusted for the same covariates: age, sex, race, and Elixhauser index (for burden of comorbidities).

The time to event was calculated starting from the date of informed consent/enrollment till the first between the date of discharge alive from the hospital or the date of death truncated at 60 days. Patients discharged alive or patients still in hospital at 60 days were censored.

We used SPSS Statistics Version 26 (IBM Corp., Armonk, NY) and the per-comparison significance was set at a two-tailed $P<0.05$.

Results

Supplemental Figure 1 below shows the relationship between baseline EAA, mortality at 60 days and baseline SOFA (Supplemental Figure 1A) or baseline MODS (Supplemental Figure 1B).

Supplemental Figures 2 and 3 show the alluvial plots for 28 and 60-day mortality respectively according to the changes of EAA, SOFA and MODS over days 0 (baseline) to 3.

Supplemental Figures 4-5-6 report the adjusted 60-day survival curves for a patients baseline EAA ≥0.6, SOFA >11, MODS >9 respectively.

In detail, adjusted 60-day survival was not different between patients with baseline EAA <0.6 compared to patients with EAA ≥0.6 (HR 1.91 with 95%CI 0.70-5.24, $P=0.21$) as reported in Supplemental Figure 4.

On the other hand, adjusted 60-day survival at 60 days was lower for patients with SOFA >11 with HR 3.10 (95%CI 1.39-6.92, $P=0.006$) as reported in Supplemental Figure 5, as well as for patients with MODS >9 with HR 9.23 (95%CI 3.41-24.97, $P<0.001$) as reported in Supplemental Figure 6.

SUPPLEMENTAL APPENDIX 4 - INDIVIDUAL ORGAN SYSTEM FAILURES AND EAA

Material and Methods

SOFA/MDOS/EAA at baseline and over days 1-2-3 in relation to 28-day mortality

SOFA/MODS/EAA mean over days 1-3 was compared to their baseline value using a paired T-test and we reported mean differences (95%CI). Mortality was compared between patients whose mean SOFA/MODS/EAA increased over days 1-3 (compared to baseline) against those whose scores did not increase. A subgroup analysis including only patients with EAA≥0.6 was also performed.

Severity of shock

For the *severity of shock*, we defined the vasopressor dose as norepinephrine equivalents as described by Kotani and colleague (2). In details, the maximum daily dose of each type of vasopressor will be recorded and then converted to norepinephrine equivalents using the following formula: Norepinephrine dose (µg/kg/min) + epinephrine dose (µg/kg/min) + 1/100×dopamine dose (µg/kg/min) + 0.06×phenylephrine dose(µg/kg/min) + 2.5×vasopressin dose (U/min) + 0.0025×angiotensin II dose (ng/kg/min) + 10×terlipressin dose (µg/kg/min) + 0.2×methylene blue dose (mg/kg/h) + 8×metaraminol dose (µg/kg/min) + 0.02×hydroxocobalamin dose (g) + 0.4×midodrine dose (µg/kg/min).

We reported the baseline vasopressor dose (day 0) and the daily dose for day 1, 2 and 3. Since many patients were off vasopressor after day 1, for the analysis we pooled together day 1-2-3 reporting the median vasopressor dose between day 1-2-3 after checking that this continuous variable was not normally distributed according to Kolmogorov-Smirnov test (P<0.001). We compared vasopressor dose at baseline from the median over days 1 to 3 using Wilcoxon paired test and we reported Hodges-Lehman Median Difference (95% confidence interval (CI)). We also compared the vasopressor dose at baseline and over days 1-3 between patients with baseline EAA ≥0.6 vs <0.6 using Mann-Whitney U Test.

Acute Kidney Injury

Acute kidney injury (AKI) was defined and staged according to serum creatinine criteria from Kidney Disease Improving Global Outcomes (KDIGO)(3), excluding patients with end-stage renal disease. We assessed the presence of AKI and its stage at baseline (day 0) and daily for day 1, 2 and 3. For the analysis we then determined the highest AKI stage over days 0 to 3.

We compared baseline EAA between patients with and without AKI using independent sample T-test and we reported the mean difference (95%CI). We determined the differences in the distribution of patients with baseline EAA < or ≥0.6 and presence of AKI using Pearson's Chi-Square. Using Chi-Square test, we also analyzed the distribution of patients with severe AKI (KDIGO stages 2 and 3) in patients with EAA< or ≥0.6, and of AKI/severe AKI in patients with or without endotoxic septic shock (ESS).

Respiratory dysfunction

Respiratory dysfunction was defined as the ratio of arterial oxygen partial pressure to fractional inspired oxygen (P/F) <300 and severe respiratory dysfunction/failure as the need of *mechanical ventilation*. We assessed the rates of respiratory dysfunction and mechanical ventilation at baseline (day 0) and daily for day 1, 2 and 3. For the analysis we then determined the presence of any respiratory dysfunction/mechanical ventilation at any time over days 0 to 3.

We compared baseline EAA between patients with and without respiratory dysfunction/mechanical ventilation using independent sample T-test and we reported the mean difference (95%CI). We determined the differences in the distribution of patients with baseline EAA < or ≥0.6 and presence of respiratory dysfunction/mechanical ventilation using Pearson's Chi-Square. Using Chi-Square test, we also analyzed the distribution of patients with respiratory dysfunction (or mechanical ventilation) in patients with or without ESS.

Neurological dysfunction

Neurological dysfunction was defined as the presence of neurological dysfunction as a Glasgow Coma Scale ≤9 at any time over days 0 to 3.

We compared baseline EAA between patients with and without neurological dysfunction using independent sample T-test and we reported the mean difference (95%CI). We determined the differences in the distribution of patients with baseline EAA < or ≥0.6 and presence of neurological dysfunction using Pearson's Chi-Square. Using Chi-Square test, we also analyzed the distribution of patients with neurological dysfunction in patients with or without ESS.

Hepatic dysfunction

Hematological dysfunction was defined as the presence of as a bilirubin ≥2 mg/dL at any time over days 0 to 3. at any time over days 0 to 3.

We compared baseline EAA between patients with and without hepatic dysfunction using independent sample T-test and we reported the mean difference (95%CI). We determined the differences in the distribution of patients with baseline EAA < or ≥0.6 and presence of hepatic dysfunction using Pearson's Chi-Square. Using Chi-Square test, we also analyzed the distribution of patients with hepatic dysfunction in patients with or without ESS.

Hematological dysfunction

Hematological dysfunction was defined as the presence of as the platelets count <100000/mcL at any time over days 0 to 3.

We compared baseline EAA between patients with and without hematological dysfunction using independent sample T-test and we reported the mean difference (95%CI). We determined the differences in the distribution of patients with baseline EAA < or ≥0.6 and presence of

hematological dysfunction using Pearson's Chi-Square. Using Chi-Square test, we also analyzed the distribution of patients with hematological dysfunction in patients with or without ESS.

Results

SOFA/MDOS/EAA at baseline and over days 1-2-3 in relation to 28-day mortality

Mean SOFA score over days 1-3 collectively was 8.3 (SD 4.5) and decreasing compared to mean SOFA at baseline (mean difference -2.0, 95%CI -2.6 to -1.4, *P*<0.001). Mean MODS over days 1-3 was 5.1 (SD 3.5), and lower than MODS at baseline (mean difference -0.8, 95%CI -1.3 to -0.3, *P*=0.003). By contrast, mean EAA over days 1-3 was 0.63 (SD 0.18) and it was not different from EAA at baseline (mean difference -0.01, 95%CI -0.05 to 0.03, *P*=0.73).

	Baseline (day 0)	*Mean over days 1 to 3*	*Mean Difference*	*P*
EAA	0.64 (SD 0.19)	0.63 (SD 0.18)	-0.01 (95%CI -0.05 to 0.03)	0.73
SOFA	10.3 (SD 3.2)	8.3 (SD 4.5)	-2.0 (95%CI -2.6 to -1.4)	<0.001
MODS	5.8 (SD 3.1)	5.1 (SD 3.5)	-0.8 (95%CI -1.3 to -0.3)	0.003

For the 20 patients, whose SOFA increased over days 1-3 compared to baseline, 11 died by day 28 (55%) while for the remaining 70 patients, 28-day mortality was 12/70 (17.1%) (*P*=0.001). Similar results were seen for the 28 patients with increased MODS, 12 died (42.9%) by day 28 compared to 11 deaths (17.7%) among the 62 patients with MODS that did not increase (*P*=0.01). Differently, 28-day mortality occurred in 10/42 (23.8%) patients with EAA increasing and 11/44 (25%) died with EAA not increasing (*P*=0.90). When this comparison was limited to patients with baseline EAA ≥0.6, 7 of 16 (43.8%) died with increasing EAA while 9 of 35 (25.7%) died with EAA stable or decreasing (*P*=0.20).

Severity of shock

Vasopressor doses at baseline had a median of 0.2 mcg/kg/min [interquartile range (IQR) 0.1-0.33] while the median over days 1-3 was lower with a value of 0.04 mcg/kg/min [IQR 0.00-0.12]. The median difference from days 1-3 to baseline was -0.14 (95%CI -0.18 to -0.11, *P*<0.001).

Patients with baseline EAA ≥0.6 had a higher vasopressor dose over days 1-3 of 0.08 [IQR 0.00-0.18] compared to patients with EAA <0.6 who had a median value of 0.02 [IQR 0.00-0.07] and with a median difference of 0.03 (95%CI 0.00 to 0.08, *P*=0.02). On the other hand, vasopressor dose at baseline did not vary according to baseline EAA: median was 0.22 [IQR 0.10-0.40] for EAA ≥0.6 while 0.17 [IQR 0.11-0.27] for EAA <0.6, with a median difference of 0.03 (95%CI -0.02 to 0.12, P=0.30).

Acute Kidney Injury

We staged AKI over days 0 to 3 in the 82 patients without end-stage renal disease.

AKI occurred in 48/82 patients (58.5%)—for most (41) patients, AKI was already present at baseline. The highest KDIGO stage over days 0-3 was stage 1 in 14 patients (17.1%), stage 2 in 9 patients (11%) and stage 3 in 25 patients (30.5%).

For the 34 patients who never developed AKI, the mean EAA at baseline was 0.60 (SD 0.22) and 14 (41.2%) of them had EAA ≥0.6 while 3 (8.8%) had EAA non-responder (NR). By contrast, for the 48 patients who developed AKI, mean EAA was 0.64 (SD 0.18) and 29 patients of them (60.4%) had EAA ≥0.6 and 1 patient had EAA value NR (2.1%). Baseline EAA mean difference between patients without and with AKI was -0.04 (95%CI -0.13 to 0.05, *P*=0.36).
Also, there was no difference among the distribution of patients with EAA < or ≥0.6 or NR (*P*=0.14) as shown in the table below:

	EAA <0.6	*EAA ≥0.6*	*EAA NR*	*Total*
no AKI	17 (50%)	14 (41.2%)	3 (8.8%)	34
AKI	18 (37.5%)	29 (60.4%)	1 (2.1%)	48
Total	35 (42.7%)	43 (52.4%)	4 (4.9%)	82

On the other hand, EAA was more likely to be ≥0.6 in patients with more severe AKI (70.6% for stage 2-3 vs 38.5% in stage 1; *P*=0.04) as shown in the table below:

	EAA <0.6	*EAA ≥0.6*	*Total*
Stage 1	8 (61.5%)	5 (38.5%)	13
Stage 2-3	10 (29.4%)	24 (70.6%)	34
Total AKI	18 (38.3%)	29 (61.7%)	47

Furthermore, patients with ESS were more likely to develop AKI (16/19, 84.2%) compared to other patients with septic shock (31/63, 50.8%, *P*=0.01). Moreover, stage 2-3 AKI occurred more frequently in 13/19 patients (68.4%) with ESS compared to 21/63 patients (33.3%) with non-ESS (*P*=0.02). See Table 2 in the main text.

Respiratory Dysfunction

A total of 69 (76.7%) patients had respiratory dysfunction at any time over days 0 to 3 (58 of them already at baseline), while 27 (30.0%) patients were under mechanical ventilation over days 0 to 3 (18 of them already at baseline).
For the 21 patients who never had respiratory dysfunction over days 0 to 3, mean EAA at baseline was 0.60 (SD 0.16) and 11 (52.4%) of them had EAA ≥0.6 while 1 (4.8%) patient had EAA NR. For the 69 patients who had respiratory dysfunction, mean EAA at baseline was 0.65 (SD 0.20) and 40 (58.0%) of them had EAA ≥0.6 while 3 (4.3%) patients had EAA NR. Baseline EAA mean difference between patients without and with respiratory dysfunction was -0.05 (95%CI -0.15 to 0.05, *P*=0.31).
EAA was ≥0.6 for 11/21 (52.4%) of patients without respiratory dysfunction, but it was not statistically different when compared to the proportion of patients with EAA ≥0.6 and respiratory dysfunction (40 patients out of 69 (58.0%), *P*=0.90).

For 63 patients who never needed mechanical ventilation over days 0 to 3, mean EAA at baseline was 0.60 (SD 0.19) and 30 (47.6%) of them had EAA ≥0.6 while 2 (3.2%) patients had EAA NR. For the 27 patients who needed mechanical ventilation, mean EAA at baseline was 0.73 (SD 0.17) and 21 (77.8%) of them had EAA ≥0.6 while 2 (7.4%) patients had EAA NR. Baseline EAA mean difference between patients without and with respiratory dysfunction was -0.13 (95%CI -0.21 to -0.04, *P*=0.005).

EAA was ≥0.6 for 30/63 (47.6%) of patients not receiving mechanical ventilation, while it was more often ≥0.6 in the 27 mechanical ventilated patients (77.8%, *P*=0.008).

All 21 patients with ESS had respiratory dysfunction and 17 (81%) required mechanical ventilation. In comparison, in the 69 patients without ESS, 48 (69.6%) had respiratory dysfunction and only 10 (14.5%) were mechanically ventilated (*P*=0.004 and <0.001 respectively). See Table 2 in the main text.

Neurological dysfunction

A total of 43 (47.8%) patients had neurological dysfunction at any time over days 0 to 3 (26 of them already at baseline).

For the 47 patients who never had neurological dysfunction over days 0 to 3, mean EAA at baseline was 0.59 (SD 0.19) and 20 (42.6%) of them had EAA ≥0.6 while 2 (4.3%) patients had EAA NR. For the 43 patients who had neurological dysfunction, mean EAA at baseline was 0.68 (SD 0.18) and 31 (72.1%) of them had EAA ≥0.6 while 2 (4.7%) patients had EAA NR. Baseline EAA mean difference between patients without and with neurological dysfunction was -0.09 (95%CI -0.17 to -0.01, *P*=0.03).

EAA was ≥0.6 for 20/47 (42.6%) of patients without neurological dysfunction, while it was more often ≥0.6 in the 43 patients with neurological dysfunction over day 0 to 3 (72.1%, *P*=0.01).

Among the 21 patients with ESS, 18 (85.7%) patients had neurological dysfunction while only 25 out of 69 (36.2%) had neurological dysfunction among non-ESS (*P*<0.001). See Table 2 in the main text.

Hepatic dysfunction

A total of 27 (30%) patients had hepatic dysfunction at any time over days 0 to 3 (22 of them already at baseline).

For the 63 patients who never had hepatic dysfunction over days 0 to 3, mean EAA at baseline was 0.62 (SD 0.19) and 33 (52.4%) of them had EAA ≥0.6 while 3 (4.8%) patients had EAA NR. For the 27 patients who had hepatic dysfunction, mean EAA at baseline was 0.67 (SD 0.19) and 18 (66.7%) of them had EAA ≥0.6 while 1 (3.7%) patient had EAA NR. Baseline EAA mean difference between patients without and with hepatic dysfunction was -0.04 (95%CI -0.13 to 0.05, *P*=0.33).

EAA was ≥0.6 for 33/63 (52.4%) of patients without hepatic dysfunction, but it was not statistically different when compared to the proportion of patients with EAA ≥0.6 and hepatic dysfunction (18 patients out of 27 (66.7%), *P*=0.45).
Among the 21 patients with ESS, 12 (57.1%) patients had hepatic dysfunction while only 15 out of 69 (21.7%) had hepatic dysfunction among non-ESS (*P*=0.002). See Table 2 in the main text.

Hematological dysfunction

A total of 33 (36.7%) patients had hematological dysfunction at any time over days 0 to 3 (15 of them already at baseline).
For the 57 patients who never had hematological dysfunction over days 0 to 3, mean EAA at baseline was 0.62 (SD 0.20) and 28 (49.1%) of them had EAA ≥0.6 while 3 (5.3%) patients had EAA NR. For the 33 patients who had hematological dysfunction, mean EAA at baseline was 0.67 (SD 0.17) and 23 (69.7%) of them had EAA ≥0.6 while 1 (3.0%) patient had EAA NR. Baseline EAA mean difference between patients without and with hematological dysfunction was -0.06 (95%CI -0.14 to 0.03, *P*=0.18).
EAA was ≥0.6 for 28/57 (49.1%) of patients without hematological dysfunction, but it was not statistically different when compared to the proportion of patients with EAA ≥0.6 and hematological dysfunction (23 patients out of 33 (69.7%), *P*=0.17).
Among the 21 patients with ESS, 15 (71.4%) patients had hematological dysfunction while only 18 out of 69 (26.1%) had hematological dysfunction among non-ESS (*P*<0.001). See Table 2 in the main text.

SUPPLEMENTAL APPENDIX 5 – SENSITIVITY ANALYSIS 1 – LACTATE > 2.0

Methods

We performed a sensitivity analysis restricted to patients that had a maximum lactate over day 0 (baseline) to 3 higher than 2.0 mmol/L.

This sensitivity analysis replicated what performed for the primary analysis, in details: we classified baseline *SOFA as ≤ or >11*, *MODS ≤ or >9*, and *EAA < or ≥0.6*. Endotoxic septic shock (ESS) was defined as a baseline EAA ≥0.6 plus either SOFA>11 or MODS>9. Our primary outcome was *28-day mortality*.

Results

A total of 53 (58.9%) patients had also lactate >2.0 mmol/L and would have met also this criterium for septic shock by Sepsis-3 (together with the hypotension/vasopressor criterion). Among the 21 patients with ESS, 17 (81%) had lactate >2.0. Daily lactate is shown in Table 1, the percentage of patients with maximum lactate >2.0 mmol/L in the whole cohort is shown as well in Table 1 while the distribution among patients with or without ESS is shown in Table 2.

Among the 53 patients with lactate >2.0 mmol/L, 28-day mortality occurred in 3/18 (16.7%) patients with a baseline EAA <0.6 while 14/33 (42.4%) died with EAA 0.6 or greater. Two patients were NR for EAA and 1 died.

EAA ≥0.6 and SOFA >11 were present together in 17 patients (32.1%) and 11 (64.7%) of these patients died by day 28. By contrast EAA ≥0.6 and SOFA ≤11 occurred in 16 (30.2%), and 3 (18.8%) died. Of the 18 remaining patients with EAA <0.6, 17 (32.1%) had SOFA ≤11 and 3 (17.6%) died. Only 1 patient (1.9%) had low EAA and high SOFA and he was alive at day 28 (overall P=0.01).

Results were qualitatively similar using MODS: all patients with MODS >9 also had EAA ≥0.6 (10 patients, 18.9%) and 28-day mortality was 80%. High EAA with MODS ≤9 occurred in 23 (43.4%) patients, 6 (26.1%) of them died by day 28. 18 patients (34%) had low MODS and EAA and only 3 (16.7%) patients died (overall P=0.002).

ESS (EAA ≥0.6 and high organ failure using either SOFA >11 or MODS >9) was present in 17 patients (32.1%) and compared to remaining patients with other forms of septic shock (non-ESS), those with ESS had significantly higher mortality (64.7% vs 19.4%, *P*=0.001) with relative risk for death of 3.33 (95%CI 1.57-7.06).

Supplemental Table 1 below shows next to each other the results of this sensitivity and of the primary analysis.

SUPPLEMENTAL APPENDIX 6 – SENSITIVITY ANALYSIS 2 – ALTERNATIVE CUTOFFS

Statistical analysis

We performed a sensitivity analysis evaluating other cutoffs for baseline SOFA and MODS. We performed receiver operating characteristic (ROC) curves to assess area under curve (AUC) and the sensitivity (Sn) and specificity (Sp) for the different cutoffs for SOFA and MODS in predicting 28-day mortality. For each cutoff we assessed the corresponding Youden index.
Then, we defined endotoxic septic shock (ESS) using the best cutoff for SOFA and MODS and we compared 28-day mortality in patients with ESS was compared to non-ESS patients using Pearson's Chi-square test and relative risk with its 95% confidence interval (CI) like in the main analysis.

Results

The AUC for baseline SOFA for 28-day mortality was 0.74 (95%CI 0.64-0.83, *P*<0.001). The table below shows the corresponding Sn, Sp and Youden index for each cutoff. Bold green font indicates the cutoff used for the main analysis (SOFA >11). Red italics indicates the best cutoff according to the highest Youden index (SOFA >12).

SOFA cutoffs	*Sensitivity*	*Specificity*	*Youden*
≥4	100.00	0.00	0.000
>4	100.00	1.49	0.015
>5	100.00	2.99	0.030
>6	100.00	13.43	0.134
>7	91.30	26.87	0.182
>8	86.96	40.30	0.273
>9	78.26	50.75	0.290
>10	60.87	65.67	0.265
**>11**	**56.52**	**79.10**	**0.356**
>12	*52.17*	*88.06*	*0.402*
>13	39.13	91.04	0.302
>14	34.78	95.52	0.303
>15	21.74	98.51	0.203
>16	21.74	100.00	0.217
>17	8.70	100.00	0.087
>18	4.35	100.00	0.044
>19	0.00	100.00	0.000

The AUC for baseline MODS for 28-day mortality was 0.70 (95%CI 0.60-0.80, *P*=0.004). The table below shows the corresponding Sn, Sp and Youden index for each cutoff. Bold green font indicates the cutoff used for the main analysis (MODS >9). Red italics indicates the best cutoff according to the highest Youden index (MODS >8).

MODS cutoffs	*Sensitivity*	*Specificity*	*Youden*
≥0	100.00	0.00	0.000
>0	100.00	1.49	0.015
>1	100.00	4.48	0.045
>2	95.65	17.91	0.136
>3	82.61	23.88	0.065
>4	78.26	44.78	0.230
>5	65.22	59.70	0.249
>6	56.52	68.66	0.252
>7	47.83	79.10	0.269
>8	*47.83*	*94.03*	*0.419*
**>9**	**39.13**	**95.52**	**0.347**
>10	34.78	97.01	0.318
>11	17.39	98.51	0.159
>12	13.04	100.00	0.130
>13	4.35	100.00	0.044
>14	0.00	100.00	0.000

We then used SOFA >12 and MODS>8 to define ESS since these were the cutoffs with the best Sn and Sp combination and consequently the highest Youden index. With these criteria, 20 (22.2%) patients had ESS. Patients with ESS had significantly higher mortality (60% vs 15.7%, P<0.001) with relative risk of 3.82 (95%CI 1.99-7.31) compared to non-ESS patients.

SUPPLEMENTAL TABLE 1 – SENSITIVITY ANALYSIS FOR LACTATE >2.0

	Sensitivity Analysis Lactate >2.0 mmol/L		*Primary Analysis All Patients*	
	Total (N=53)	28-day Mortality	Total (N=90)	28-day Mortality
EAA NR	2 (3.8%)	1 (50%)	4 (4.4%)	2 (50%)
EAA ≥0.6 and SOFA >11	17 (32.1%)	11 (64.7%)	20 (23.3%)	12 (60%)
EAA ≥0.6 and SOFA ≤11	16 (30.2%)	3 (18.8%)	31 (36.0%)	4 (12.9%)
EAA <0.6 and SOFA >11	1 (1.9%)	0 (0%)	6 (7.0%)	0 (0%)
EAA <0.6 and SOFA ≤11	17 (32.1%)	3 (17.6%)	29 (33.7%)	5 (17.2%)
EAA ≥0.6 and MODS >9	10 (18.9%)	8 (80%)	12 (13.3%)	9 (75%)
EAA ≥0.6 and MODS ≤9	23 (43.4%)	6 (26.1%)	39 (45.3%)	7 (17.9%)
EAA <0.6 and MODS >9	0 (0%)	0 (0%)	0 (0%)	0 (0%)
EAA <0.6 and MODS ≤9	18 (34%)	3 (16.7%)	35 (38.9%)	5 (14.3%)
ESS	17 (32.1%)	11 (64.7%)	21 (23.3%)	12 (57.1%)
Non-ESS	36 (67.9%)	7 (19.4%)	69 (76.7%)	11 (15.9%)

Categorical variables are presented as numbers (%).
Abbreviations: EAA = endotoxin activity assay; MODS = Multiple Organ Dysfunction Score; NR = non-responder; SOFA = Sequential Organ Failure Assessment.

For patients with Lactate > 2 mmol/L (N=53), ESS (EAA≥0.6 together with SOFA>11 or MODS>9) 28-day mortality was 64.7% vs 19.4% for non-ESS, (P=0.001) with relative risk for death of 3.33 (95%CI 1.57-7.06).
For the entire cohort (N=90), ESS (EAA≥0.6 together with SOFA>11 or MODS>9) 28-day mortality was 57.1% vs 15.9%, for non-ESS, (P<0.001), with a relative risk for death of 3.58 (95%CI 1.86-6.91).

SUPPLEMENTAL TABLE 2 – CLINICAL PHENOTYPES FOR SEPSIS

	Total *N. 90*	*Alpha* *N. 3*	*Beta* *N. 63*	*Gamma* *N. 13*	*Delta* *N. 11*
EAA baseline	0.64 (0.19)	0.65 (0.31)	0.61 (0.19)	0.68 (0.2)	0.73 (0.16)
EAA baseline ≥0.6	51 (56.7%)	2 (66.7%)	35 (55.6%)	6 (46.2%)	8 (72.7%)
EAA baseline NR	4 (4.4%)	0 (0%)	1 (1.6%)	2 (15.4%)	1 (9.1%)
SOFA baseline	10.3 (3.2)	8.7 (2.9)	10.0 (2.9)	8.6 (2.4)	14.2 (3.1)
SOFA baseline >11	27 (30%)	1 (33.3%)	16 (25.4%)	1 (7.7%)	9 (81.8%)
MODS baseline	5.8 (3.1)	4.3 (3.2)	5.6 (3)	4.8 (2.2)	9.0 (2.9)
MODS baseline >9	12 (13.3%)	0 (0%)	8 (12.7%)	0 (0%)	4 (36.4%)
Mortality at 28 days	23 (25.6%)	0 (0%)	16 (25.4%)	2 (15.4%)	5 (45.5%)
ESS	21 (23.3%)	0 (0%)	13[a] (20.6%)	1 (7.7%)	7 (63.6%)

Continuous variables are expressed as means (standard deviation). Categorical variables are presented as numbers (%).

Abbreviations: AKI = acute kidney injury; EAA = endotoxin activity assay; ESS = endotoxic septic shock; MODS = Multiple Organ Dysfunction Score; NR = non-responder; SOFA = Sequential Organ Failure Assessment.

SUPPLEMENTAL FIGURE 1 - BASELINE EAA, SOFA/MODS AND 60-DAY MORTALITY

In the figure each patient is represented by a dot/square according to his baseline (day 0) values for EAA and organ failure scores (Top: Figure A for SOFA; bottom: Figure B for MODS). Full black circles are for patients who died before 60 days while empty squares are for patients alive at 60 days (or discharged alive before day 60). Solid black lines represent the cutoff values of 11 for SOFA (A), of 9 for MODS (B) and of 0.60 for EAA.

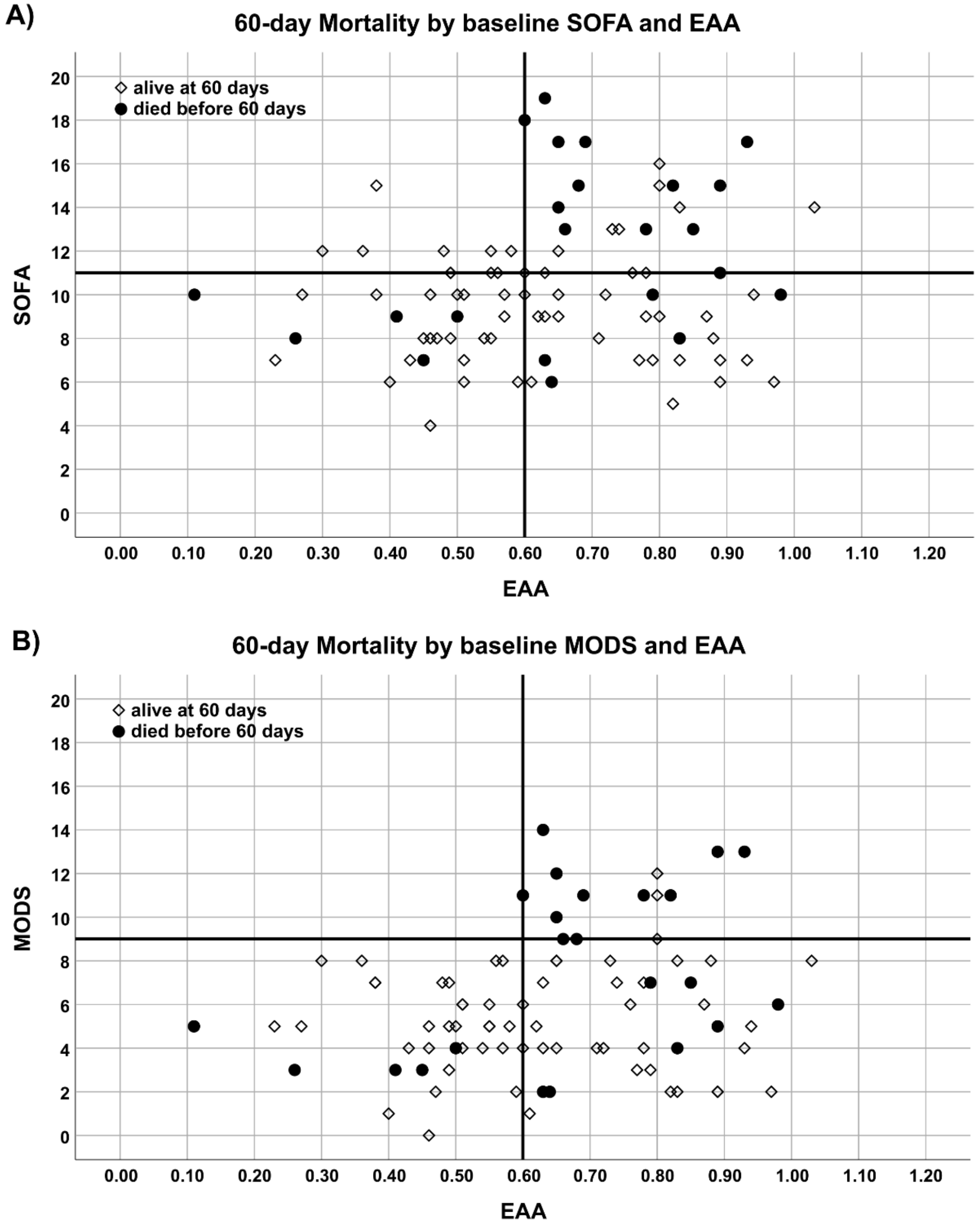

Abbreviations: EAA = endotoxin activity assay; MODS = Multiple Organ Dysfunction Score; NR = non-responder; SOFA = Sequential Organ Failure Assessment.

SUPPLEMENTAL FIGURE 2 – ALLUVIAL PLOTS FOR 28-DAY MORTALITY

The alluvial plots show for each patient how EAA (panel A), SOFA (panel B) and MODS (panel C) changed over days 0 (baseline), 1, 2 and 3. Colors reflect 28-day mortality: in light blue patients who were alive at 28 days (or discharged alive before day 28), in red patients who died before 28 days.

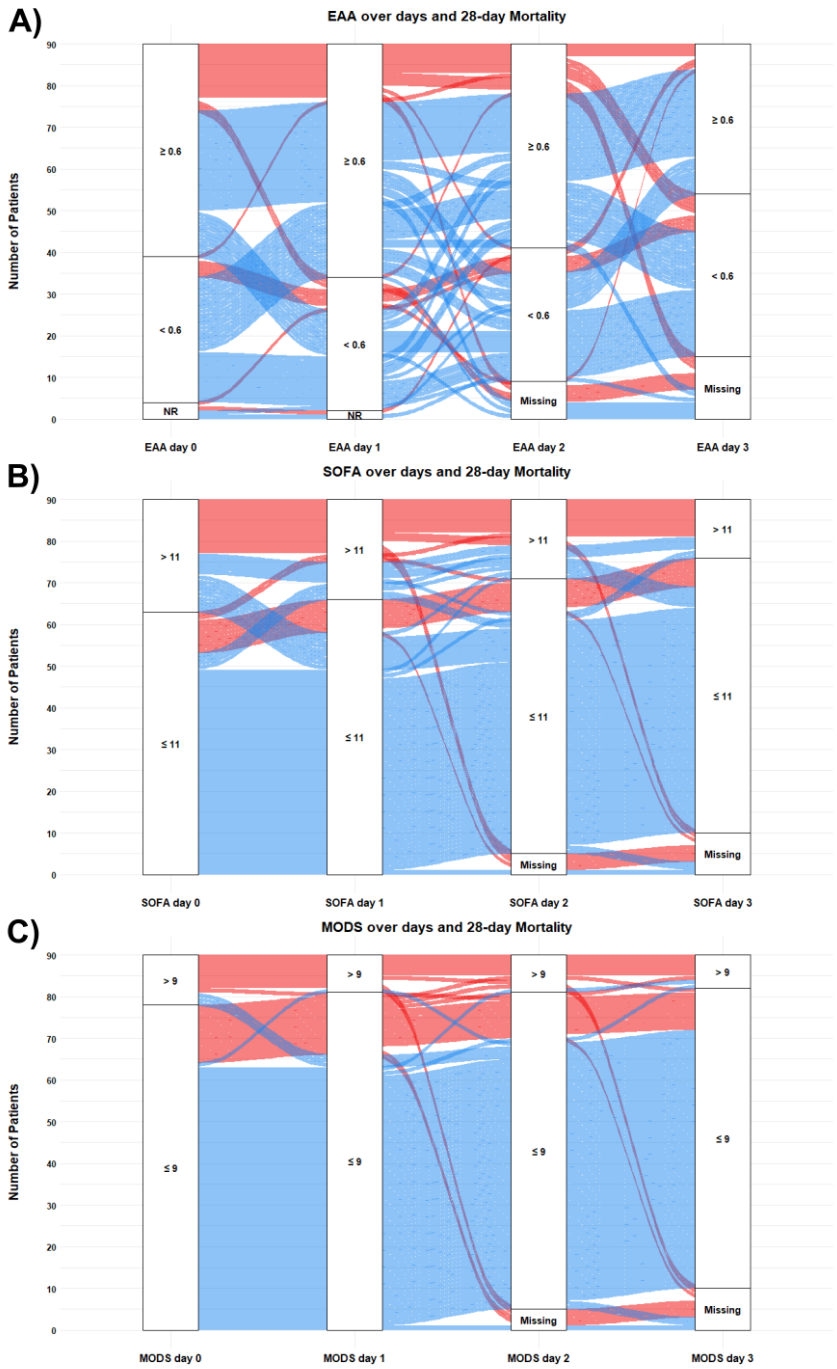

Abbreviations: EAA = endotoxin activity assay; MODS = Multiple Organ Dysfunction Score; SOFA = Sequential Organ Failure Assessment.

SUPPLEMENTAL FIGURE 3 – ALLUVIAL PLOTS FOR 60-DAY MORTALITY

The alluvial plots show for each patient how EAA (panel A), SOFA (panel B) and MODS (panel C) changed over days 0 (baseline), 1, 2 and 3. Colors reflect 28-day mortality: in light blue patients who were alive at 60 days (or discharged alive before day 60), in red patients who died before 60 days.

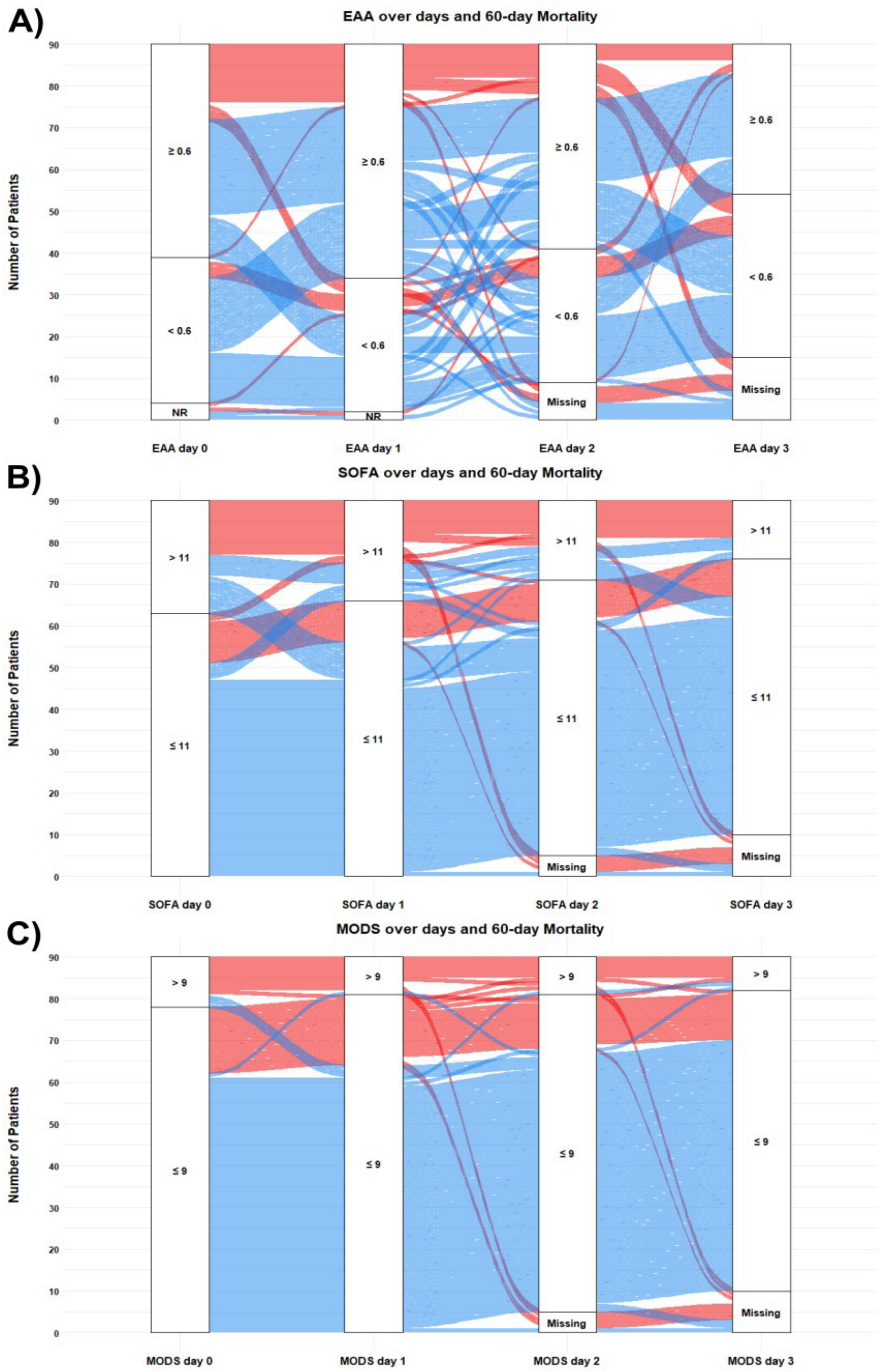

Abbreviations: EAA = endotoxin activity assay; MODS = Multiple Organ Dysfunction Score; SOFA = Sequential Organ Failure Assessment.

SUPPLEMENTAL FIGURE 4 – ADJUSTED 60-DAY SURVIVAL BY BASELINE EAA

Shown are the adjusted 60-day survival curves obtained from Cox proportional hazard model for patients with baseline (day 0) EAA <0.6 (in light blue) and ≥0.6 (in red).
Numbers of patients at risk of death are shown beneath the figure.
The model was adjusted for age, sex, race, and Elixhauser index (for burden of comorbidities).
Four patients (out of 90 patients, 4.4%) had baseline EAA result of "non-responder", and they were excluded from this analysis (2 died before discharge, 1 was discharged alive at day 7, 1 was still admitted and alive at day 60).
See Supplemental Appendix 3 for details.

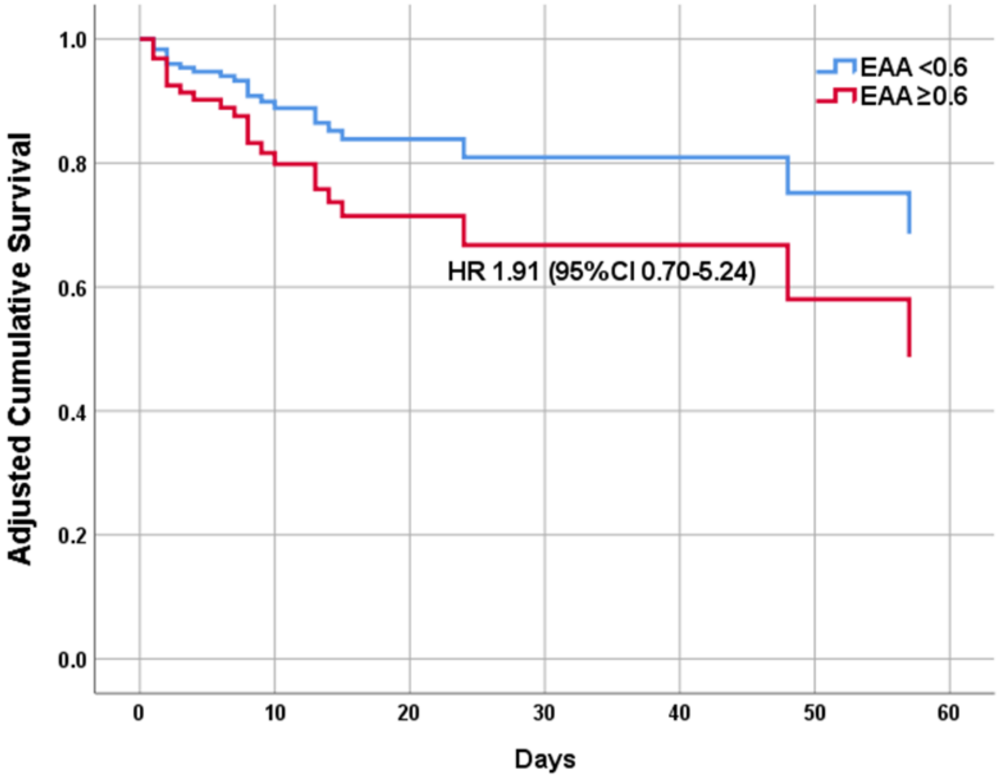

NUMBER AT RISK

**EAA <0.6**							
	35	11	5	3	2	2	0
**EAA ≥ 0.6**							
	51	28	15	8	6	5	0

Abbreviations: CI = confidence interval; EAA = endotoxin activity assay; HR = hazard ratio.

SUPPLEMENTAL FIGURE 5 – ADJUSTED 60-DAY SURVIVAL BY BASELINE SOFA

Shown are the adjusted 60-day survival curves obtained from Cox proportional hazard model for patients with baseline (day 0) SOFA score ≤11 (in light blue) and >11 (in red).
Numbers of patients at risk of death are shown beneath the figure.
The model was adjusted for age, sex, race, and Elixhauser index (for burden of comorbidities).
See Supplemental Appendix 3 for details.

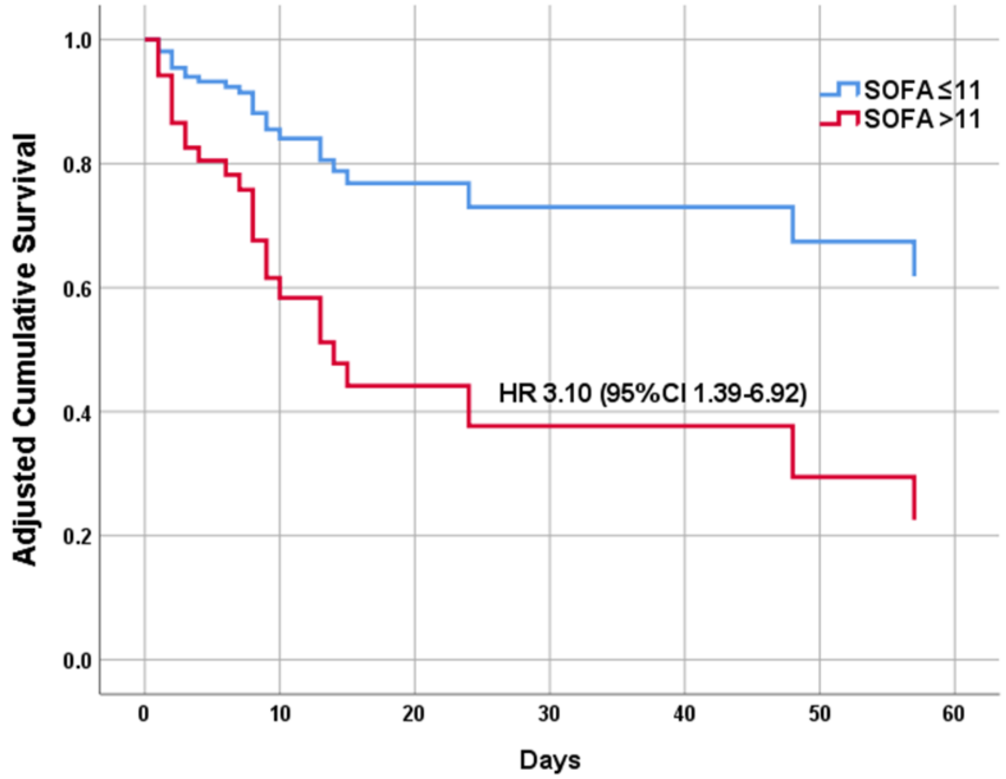

NUMBER AT RISK

SOFA ≤11	63	32	16	8	6	5	0
SOFA >11	27	8	5	4	3	3	0

Abbreviations: CI = confidence interval; HR = hazard ratio; SOFA = Sequential Organ Failure Assessment.

SUPPLEMENTAL FIGURE 6 – ADJUSTED 60-DAY SURVIVAL BY BASELINE MODS

Shown are the adjusted 60-day survival curves obtained from Cox proportional hazard model for patients with baseline (day 0) MODS score ≤9 (in light blue) and >9 (in red).
Numbers of patients at risk of death are shown beneath the figure.
The model was adjusted for age, sex, race, and Elixhauser index (for burden of comorbidities).
See Supplemental Appendix 3 for details.

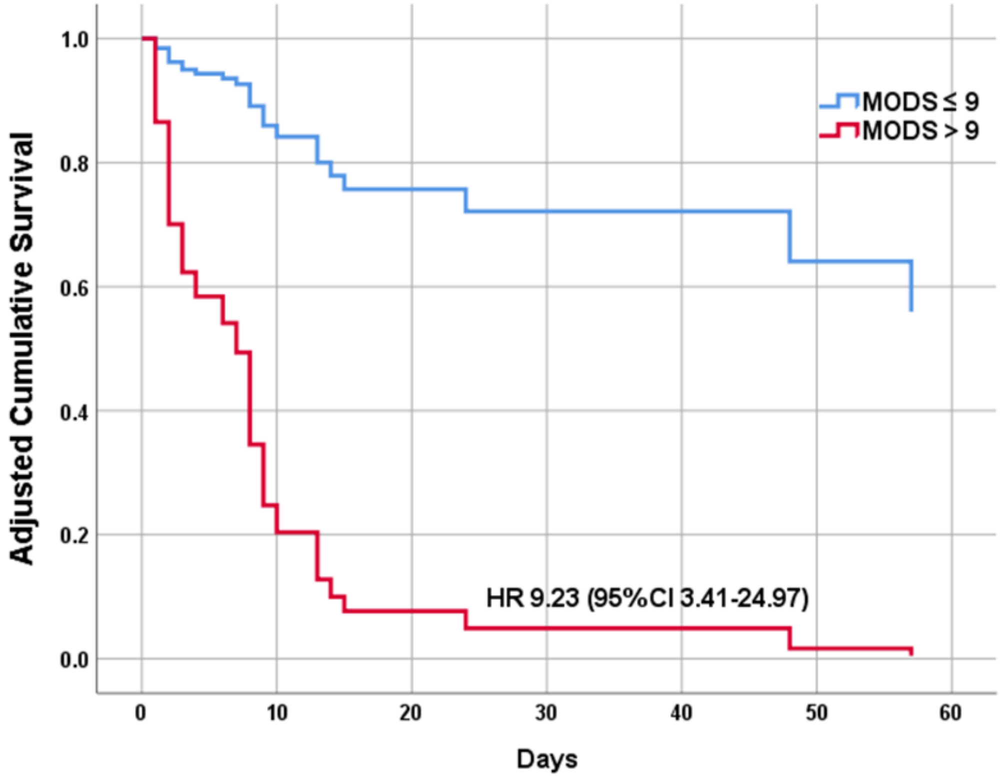

NUMBER AT RISK

	0	10	20	30	40	50	60
MODS ≤ 9	78	39	20	12	9	8	0
MODS > 9	12	1	1	0	0	0	0

Abbreviations: CI = confidence interval; HR = hazard ratio; MODS = Multiple Organ Dysfunction Score.

SUPPLEMENTAL MATERIAL REFERENCES

1. Singer M, Deutschman CS, Seymour CW, et al: The Third International Consensus Definitions for Sepsis and Septic Shock (Sepsis-3). *JAMA* 2016; 315(8):801-810
2. Kotani Y, Di Gioia A, Landoni G, et al: An updated "norepinephrine equivalent" score in intensive care as a marker of shock severity. *Crit Care* 2023; 27(1):29
3. Kidney Disease: Improving Global Outcomes (KDIGO) Acute Kidney Injury Work Group: KDIGO Clinical Practice Guideline for Acute Kidney Injury. *Kidney Int Suppl* 2012; 2(1):1-138
